# Almost sure central limit theorem for self-normalized products of the some partial sums of $\rho^{-}$-mixing sequences

**DOI:** 10.1186/s13660-018-1835-3

**Published:** 2018-09-14

**Authors:** Xili Tan, Wei Liu

**Affiliations:** Department of Mathematics and Statistics, Beihua University, Jilin, China

**Keywords:** 60F15, Almost sure central limit theorem, $\rho^{-}$-Mixing sequence, Self-normalized, Products of the some partial sums

## Abstract

Let $\{X, X_{n}\}_{n\in N}$ be a strictly stationary $\rho^{-}$-mixing sequence of positive random variables, under the suitable conditions, we get the almost sure central limit theorem for the products of the some partial sums $({\frac{\prod_{i=1}^{k}S_{k,i}}{(k-1)^{n}\mu ^{n}} )^{\frac{\mu}{\beta V_{k}}} }$, where $\beta>0$ is a constant, and ${\mathrm{E}}(X)=\mu$, $S_{k,i}=\sum_{j=1}^{k}X_{j}-X_{i}$, $1\le i\le k$, $V_{k}^{2}=\sum_{i=1}^{k}(X_{i}-\mu)^{2}$.

## Introduction and main result

In 1988, Brosamler [1] and Schatte [2] proposed the almost sure central limit theorem (ASCLT) for the sequence of i.i.d. random variables. On the basis of i.i.d., Khurelbaatar and Grzegorz [3] got the ASCLT for the products of the some partial sums of random variables. In 2008, Miao [4] gave a new form of ASCLT for products of some partial sums.

### Theorem A

([4])

*Let* $\{X, X_{n}\}_{n\in N}$ *be a sequence of i*.*i*.*d*. *positive square integrable random variables with* ${\mathrm{E}}(X_{1})=\mu$, $\operatorname{Var}(X_{1})=\sigma^{2}>0$ *and the coefficient of variation* $\gamma=\frac{\sigma}{\mu}$. *Denote the* $S_{k,i}=\sum_{j=1}^{k}X_{j}-X_{i}$, $1\leq i\leq k$. *Then*, *for* $\forall x \in R$,

$$\lim_{N\to\infty}\frac{1}{\log N}\sum_{n=1}^{N} \frac{1}{n}\mathrm{I} \biggl[ \biggl(\frac{\prod_{k=1}^{n}S_{n,k}}{(n-1)^{n}\mu^{n}} \biggr)^{\frac {1}{\gamma\sqrt{n} }} \le{x} \biggr]=F(x)\quad \textit{a.s.}, $$

*where* $F(\cdot)$ *is the distribution function of the random variables* $e^{\mathscr {N}}$, ${\mathscr {N}}$ *is a standard normal random variable*.

For random variables *X*, *Y*, define

$$\rho^{-}(X,Y)=0\vee\sup\frac{\operatorname{Cov}(f(X), g(Y))}{ (\operatorname{Var}f(X))^{\frac{1}{2}}(\operatorname{Var}g(Y))^{\frac{1}{2}}}, $$

where the sup is taken over all $f,g\in \mathscr {C}$ such that $\mathrm{E}(f(X))^{2}<\infty$ and $\mathrm{E}(g(Y))^{2}<\infty$, and $\mathscr {C}$ is a class of functions which are coordinatewise increasing.

### Definition

([5])

A sequence $\{X, X_{n}\}_{n\in N}$ is called $\rho^{-}$-mixing, if

$$\rho^{-}(s)=\sup\bigl\{ \rho^{-}(S,T); S,T\subset{N}, \operatorname{dist}(S,T)\geq{s}\bigr\} \rightarrow{0}, \quad s\rightarrow \infty, $$

where

$$\rho^{-}(S,T)=0\vee{\sup}\biggl\{ \frac{\operatorname{Cov}\{f(X_{i},i\in {S}),g(X_{j},j\in{T})\}}{ \sqrt{\operatorname{Var}\{f(X_{i},i\in{S})\}\operatorname{Var}\{g(X_{j},j\in{T})\}} }, f,g\in{ \mathscr {C}}\biggr\} , $$

$\mathscr {C}$ is a class of functions which are coordinatewise increasing.

The precise definition of $\rho^{-}$-mixing random variables was introduced initially by Zhang and Wang [5] in 1999. Obviously, $\rho^{-}$-mixing random variables include NA and $\rho ^{*}$-mixing random variables, which have a lot of applications, their limit properties have aroused wide interest recently, and a lot of results have been obtained by many authors. In 2005, Zhou [6] proved the almost central limit theorem of the $\rho^{-}$-mixing sequence. The almost sure central limit theorem for products of the partial sums of $\rho^{-}$-mixing sequences was given by Tan [7] in 2012. Because the denominator of the self-normalized partial sums contains random variables, this brings about difficulties to the study of the self-normalized form limit theorem of the $\rho ^{-}$-mixing sequence. At present, there are very few results of this kind. In this paper, we extend Theorem A, and get the almost sure central limit theorem for self-normalized products of the some partial sums of $\rho^{-}$-mixing sequences.

Throughout this paper, $a_{n}\sim b_{n} $ means $\lim_{n\to\infty }\frac{a_{n}}{b_{n} }=1$, and *C* denotes a positive constant, which may take different values whenever it appears in different expressions, and $\log x=\ln(x\vee e)$. We assume $\{X, X_{n}\}_{n\in N}$ is a strictly stationary sequence of $\rho^{-}$-mixing random variables, and we denote $Y_{i}=X_{i}-\mu$.

For every $1\leq i\leq k\leq n$, define

$$\begin{aligned}& \bar{Y}_{ni}= -\sqrt{n}\mathrm{I}(Y_{i}< - \sqrt{n})+Y_{i}\mathrm{I}\bigl( \vert Y_{i} \vert \le \sqrt{n} \bigr)+\sqrt{n}\mathrm{I}(Y_{i}>\sqrt{n}), \\& {T}_{k,n}=\sum_{i=1}^{k} \bar{Y}_{ni},\qquad V_{n}^{2}=\sum _{i=1}^{n}Y_{i}^{2},\qquad \bar{V}_{n}^{2}=\sum_{i=1}^{n} \bar{Y}_{ni}^{2}, \\& \bar{V}_{n,1}^{2}= \sum_{i=1}^{n}\bar{Y}_{ni}^{2} \mathrm{I}(Y_{i}\geq0),\qquad \bar{V}_{n,2}^{2}=\sum _{i=1}^{n}\bar{Y}_{ni}^{2} \mathrm{I}(Y_{i}< 0), \\& \sigma_{n}^{2}=\operatorname{Var}({T}_{n,n}), \qquad \delta_{n}^{2}=\mathrm{E}\bigl(\bar {Y}_{n1}^{2}\bigr),\qquad \delta_{n,1}^{2}= \mathrm{E}\bar{Y}_{n1}^{2}\mathrm{I}(Y_{1}\geq0), \qquad \delta_{n,2}^{2}=\mathrm{E}\bar{Y}_{n1}^{2} \mathrm{I}(Y_{1}< 0), \end{aligned}$$

apparently, $\delta_{n}^{2}=\delta_{n,1}^{2}+\delta_{n,2}^{2}$, $\mathrm{E}(\bar{V}_{n}^{2})=n\delta_{n}^{2}=n\delta_{n,1}^{2}+n\delta _{n,2}^{2}$.

Our main theorem is as follows.

### Theorem 1

*Let* $\{X, X_{n}\}_{n\in N}$ *be a strictly stationary* $\rho^{-}$-*mixing sequence of positive random variables with* $\mathrm{E}X=\mu>0$, *and for some* $r>2$, *we have* $0<\mathrm{E}|X|^{r}<\infty$. *Denote* $S_{k,i}=\sum_{j=1}^{k}X_{j}-X_{i}$, $1\leq i\leq k$ *and* $Y=X-\mu$. *Suppose that*

(a_1_): $\mathrm{E}v(Y^{2}\mathrm{I}(Y\geq0))>0$, $\mathrm{E}(Y^{2}\mathrm{I}(Y<0))>0$,
(a_2_): $\sigma_{1}^{2}=\mathrm{E}X_{1}^{2}+2\sum_{k=2}^{\infty}\operatorname{Cov}(X_{1},X_{k})>0$, $\sum_{k=2}^{\infty}|\operatorname{Cov}(X_{1},X_{k})|<\infty$,
(a_3_): $\sigma_{k}^{2}\sim\beta^{2}k\delta_{k}^{2}$, *for some* $\beta>0$,
(a_4_): $\rho^{-}(n)=O(\log^{-\delta}n)$, $\exists\delta>1$.

*Suppose* $0\leq\alpha<\frac{1}{2}$, *and let*

1 $$ d_{k}=\frac{\exp(\log^{\alpha}k)}{k},\qquad D_{n}=\sum _{k=1}^{n}d_{k}, $$

*then*, *for* $\forall x \in R$, *we have*

2 $$ \lim_{n\to\infty}\frac{1}{D_{n}}\sum_{k=1}^{n}d_{k} \mathrm{I} \biggl[ \biggl(\frac{\prod_{i=1}^{k}S_{k,i}}{(k-1)^{k}\mu^{k}} \biggr)^{\frac{\mu }{\beta V_{k} }}\le{x} \biggr]=F(x)\quad \textit{a.s.}, $$

*where* $F(\cdot)$ *is the distribution function of the random variables* $e^{\mathscr {N}}$, $\mathscr {N}$ *is a standard normal random variable*.

### Corollary 1

*By* [8], (2) *remains valid if we replace the weight sequence* $\{d_{k},k\geq1\}$ *by any* $\{ d_{k}^{*},k\geq1\}$ *such that* $0\leq d_{k}^{*}\leq d_{k}$, $\sum_{k=1}^{\infty}d_{k}^{*}=\infty$.

### Corollary 2

*If* $\{X_{n}, n\ge1\}$ *is a sequence of strictly stationary independent positive random variables then one has* (a_3_) *and* $\beta=1$.

## Some lemmas

We will need the following lemmas.

### Lemma 2.1

([7])

*Let* $\{X, X_{n}\}_{n\in N}$ *be a strictly stationary sequence of* $\rho^{-}$-*mixing random variables with* $\mathrm{E}X_{1}=0$, $0<\mathrm{E}X_{1}^{2}<\infty$, $\sigma_{1}^{2}=\mathrm{E}X_{1}^{2}+2\sum_{k=2}^{\infty}\operatorname{Cov}(X_{1},X_{k})>0$ *and* $\sum_{k=2}^{\infty }|\operatorname{Cov}(X_{1},X_{k})|<\infty$, *then*, *for* $0< p<2$, *we have*

$$\frac{S_{n}}{n^{\frac{1}{p}}}\rightarrow0 ,\quad \textit{a.s.}, n\rightarrow\infty. $$

### Lemma 2.2

([9])

*Let* $\{X, X_{n}\}_{n\in N}$ *be a sequence of* $\rho^{-}$-*mixing random variables*, *with*

$$\mathrm{E}X_{n}=0,\qquad \mathrm{E}|X_{n}|^{q}< \infty,\quad \forall{n}\geq1, q\geq2, $$

*then there is a positive constant* $C=C(q, \rho^{-}(\cdot))$ *only depending on* *q* *and* $\rho^{-}(\cdot)$ *such that*

$$\mathrm{E}\Bigl(\max_{1\leq{j}\leq{n}}|S_{j}|^{q} \Bigr)\leq{C}\Biggl\{ \sum_{i=1}^{n} \mathrm{E}|X_{i}|^{q}+ \Biggl(\sum _{i=1}^{n}\mathrm{E}X_{i}^{2} \Biggr)^{\frac{q}{2}}\Biggr\} . $$

### Lemma 2.3

([10])

*Suppose that* $f_{1}(x)$ *and* $f_{2}(y)$ *are real*, *bounded*, *absolutely continuous functions on* *R* *with* $|f'_{1}(x)|\leq C_{1}$ *and* $|f'_{2}(y)|\leq C_{2}$, *then*, *for any random variables* *X* *and* *Y*,

$$\bigl\vert \operatorname{Cov}\bigl(f_{1}(X), f_{2}(Y) \bigr) \bigr\vert \leq C_{1}C_{2}\bigl\{ - \operatorname{Cov}(X,Y)+8\rho ^{-}(X,Y)\|X\|_{2,1}\|Y \|_{2,1}\bigr\} , $$

*where* $\|X\|_{2,1}=\int_{0}^{\infty} (P(|X|>x) )^{\frac {1}{2}}\,dx$.

### Lemma 2.4

*Let* $\{\xi, \xi_{n}\}_{n\in N}$ *be a sequence of uniformly bounded random variables*. *If* $\exists\delta>1$, $\rho ^{-}(n)=O(\log^{-\delta}n)$, *there exist constants* $C>0$ *and* $\varepsilon>0$, *such that*

3 $$ \vert \mathrm{E}\xi_{k}\xi_{l} \vert \leq C\biggl( \rho^{-}(k)+\biggl(\frac{k}{l}\biggr)^{\varepsilon }\biggr), \quad 1\leq2k< l, $$

*then*

$$\lim_{n\rightarrow\infty}\frac{1}{D_{n}}\sum_{k=1}^{n}d_{k} \xi_{k}=0, \quad \textit{a.s.} $$

### Proof

See the proof of Theorem 1 in [7]. □

### Lemma 2.5

*If the assumptions of Theorem *1 *hold*, *then*

4 $$\begin{aligned}& \lim_{n\to\infty}\frac{1}{D_{n}}\sum_{k=1}^{n}d_{k} \mathrm{I} \biggl[\frac {{T}_{k,k}-\mathrm{E}({T}_{k,k})}{\beta\delta_{k}\sqrt{k}}\le{x} \biggr]=\Phi(x) \quad \textit{a.s.}, \forall x \in R, \end{aligned}$$

5 $$\begin{aligned}& \lim_{n\to\infty}\frac{1}{D_{n}}\sum_{k=1}^{n}d_{k} \biggl[f \biggl(\frac {\bar{V}_{k,l}^{2}}{k\delta_{k,l}^{2}} \biggr)-\mathrm{E}f \biggl(\frac{\bar {V}_{k,l}^{2}}{k\delta_{k,l}^{2}} \biggr) \biggr]=0 \quad \textit{a.s.}, l=1,2, \end{aligned}$$

*where* $d_{k}$ *and* $D_{k}$ *is defined as* (1) *and* *f* *is real*, *bounded*, *absolutely continuous function on R*.

### Proof

Firstly, we prove (4), by the property of $\rho ^{-}$-mixing sequence, we know that $\{\bar{Y}_{ni}\}_{n\geq1,i\leq n}$ is a $\rho^{-}$-mixing sequence; using Lemma 2.1 in [7], the condition (a_2_), (a_3_), and $\beta>0$, $\delta_{k}^{2}\rightarrow\mathrm{E}Y^{2}>0$, it follows that

$$\frac{{T}_{k,k}-\mathrm{E}({T}_{k,k})}{\beta\delta_{k}\sqrt{{k}}} \stackrel{\mathrm{d}}{\rightarrow} \mathscr {N},\quad k \rightarrow\infty, $$

hence, for any $g(x)$ which is a bounded function with bounded continuous derivative, we have

$$\mathrm{E}g \biggl(\frac{{T}_{k,k}-E({T}_{k,k})}{\beta\delta_{k}\sqrt {{k}}} \biggr)\rightarrow\mathrm{E}g(\mathscr {N}), \quad k\rightarrow\infty, $$

by the Toeplitz lemma, we get

$$\lim_{n\to\infty}\frac{1}{D_{n}}\sum_{k=1}^{n}d_{k} \mathrm{E} \biggl[g \biggl(\frac{{T}_{k,k}-\mathrm{E}({T}_{k,k})}{\beta\delta_{k}\sqrt{k}} \biggr) \biggr]=\mathrm{E}\bigl(g( \mathscr {N})\bigr). $$

On the other hand, from Theorem 7.1 of [11] and Sect. 2 of [12], we know that (4) is equivalent to

$$\lim_{n\to\infty}\frac{1}{D_{n}}\sum_{k=1}^{n}d_{k}g \biggl(\frac {{T}_{k,k}-\mathrm{E}({T}_{k,k})}{\beta\delta_{k}\sqrt{k}} \biggr)=\mathrm{E}\bigl(g(\mathscr {N})\bigr)\quad \mbox{a.s.}, $$

hence, to prove (4), it suffices to prove

6 $$ \lim_{n\to\infty}\frac{1}{D_{n}}\sum_{k=1}^{n}d_{k} \biggl[g \biggl(\frac {{T}_{k,k}-\mathrm{E}({T}_{k,k})}{\beta\delta_{k}\sqrt{k}} \biggr)-\mathrm{E} \biggl(g\frac{{T}_{k,k}-\mathrm{E}({T}_{k,k})}{\beta\delta_{k}\sqrt {k}} \biggr) \biggr]=0 \quad \mbox{a.s.}, $$

noting that

$$\xi_{k}=g \biggl(\frac{{T}_{k,k}-\mathrm{E}({T}_{k,k})}{\beta\delta_{k}\sqrt {k}} \biggr)-\mathrm{E} \biggl(g \biggl(\frac{{T}_{k,k}-\mathrm{E}({T}_{k,k})}{\beta\delta_{k}\sqrt{k}} \biggr) \biggr), $$

for every $1\leq2k< l$, we have

7 $$\begin{aligned} \vert \mathrm{E}\xi_{k}\xi_{l} \vert =& \biggl\vert \operatorname{Cov}\biggl(g\biggl(\frac {{T}_{k,k}-\mathrm{E}{T}_{k,k}}{\beta\delta_{k}\sqrt{k}}\biggr),g\biggl( \frac {{T}_{l,l}-\mathrm{E}{T}_{l,l}}{\beta \delta_{l}\sqrt{l}}\biggr)\biggr) \biggr\vert \\ \leq& \biggl\vert \operatorname{Cov}\biggl(g\biggl(\frac{{T}_{k,k}-\mathrm{E}{T}_{k,k}}{\beta \delta_{k}\sqrt{k}} \biggr),g\biggl(\frac{{T}_{l,l}-\mathrm{E}{T}_{l,l}}{\beta \delta_{l}\sqrt{l}}\biggr)-g\biggl(\frac{{T}_{l,l}-\mathrm{E}{T}_{l,l}-({T}_{2k,l}-\mathrm{E}{T}_{2k,l})}{\beta \delta_{l}\sqrt{l}}\biggr)\biggr) \biggr\vert \\ &{}+ \biggl\vert \operatorname{Cov}\biggl(g\biggl(\frac{{T}_{k,k}-\mathrm{E}{T}_{k,k}}{\beta \delta_{k}\sqrt{k}} \biggr), g\biggl(\frac{{T}_{l,l}-\mathrm{E}{T}_{l,l}-({T}_{2k,l}-\mathrm{E}{T}_{2k,l})}{\beta\delta_{l}\sqrt{l}}\biggr)\biggr) \biggr\vert \\ =&I_{1}+I_{2}. \end{aligned}$$

First we estimate $I_{1}$; we know that *g* is a bounded Lipschitz function, i.e., there exists a constant *C* such that

$$\bigl\vert g(x)-g(y) \bigr\vert \leq C|x-y| $$

for any $x, y\in R$, since $\{\bar{Y}_{ni}\}_{n\geq1,i\leq n}$ also is a $\rho^{-}$-mixing sequence; we use the condition $\delta_{l}^{2}\rightarrow\mathrm{E}(Y^{2})<\infty $, $l\rightarrow\infty$, and Lemma 2.2, to get

8 $$\begin{aligned} I_{1} \leq& C\frac{\mathrm{E}|{T}_{2k,l}-\mathrm{E}{T}_{2k,l}|}{\sqrt{l}}\leq C \frac{\sqrt{\mathrm{E}({T}_{2k,l}-\mathrm{E}{T}_{2k,l})^{2}}}{\sqrt {l}} \\ \leq& \frac{C}{\sqrt{l}}\sqrt{ \sum_{i=1}^{2k} \mathrm{E}\bar{Y}_{l,i}^{2}}\leq \frac{C}{\sqrt{l}}\sqrt { \sum_{i=1}^{2k} \mathrm{E}Y^{2}}\leq C\biggl(\frac{k}{l}\biggr)^{\frac{1}{2}}. \end{aligned}$$

Next we estimate $I_{2}$; by Lemma 2.2, we have

$$\begin{aligned} \operatorname{Var} \biggl(\frac{{T}_{k,k}-\mathrm{E}{T}_{k,k}}{\beta\delta_{k}\sqrt {k}} \biggr)&\leq\frac{C}{k} \operatorname{Var}({T}_{k,k}-\mathrm{E} {T}_{k,k}) \\ &\leq\frac{C}{k}\sum_{i=1}^{k} \mathrm{E}(\bar{Y}_{ki}-\mathrm{E}\bar {Y}_{ki})^{2} \leq\frac{C}{k}\sum_{i=1}^{k} \mathrm{E}(\bar {Y}_{ki})^{2}\leq\frac{C}{k} \cdot k \leq C \end{aligned}$$

and

$$\begin{aligned} \begin{aligned} \operatorname{Var} \biggl(\frac{{T}_{l,l}-\mathrm{E}{T}_{l,l}-({T}_{2k,l}-\mathrm{E}{T}_{2k,l})}{\beta \delta_{l}\sqrt{l}} \biggr)&\leq\frac{C}{l} \operatorname{Var}\bigl({T}_{l,l}-\mathrm{E} {T}_{l,l}-({T}_{2k,l}- \mathrm{E} {T}_{2k,l})\bigr) \\ &\leq\frac{C}{l}\sum_{i=2k+1}^{l} \mathrm{E}(\bar{Y}_{li}-\mathrm{E}\bar {Y}_{li})^{2}\leq\frac{C}{l}\Biggl(\sum_{i=1}^{l} \mathrm{E}\bar {Y}_{li}^{2}\Biggr) \\ &\leq\frac{C}{l} \cdot l \leq C . \end{aligned} \end{aligned}$$

By the definition of a $\rho^{-}$-mixing sequence, $\mathrm{E}Y^{2}<\infty $, and Lemma 2.3, we have

$$\begin{aligned} I_{2} \leq&\biggl( -\operatorname{Cov} \biggl(\frac{{T}_{k,k}-\mathrm{E}{T}_{k,k}}{\beta \delta_{k}\sqrt{k}}, \frac{{T}_{l,l}-\mathrm{E}{T}_{l,l}-({T}_{2k,l}-\mathrm{E}{T}_{2k,l})}{\beta \delta_{l}\sqrt{l}}\biggr) \\ &{}+8\rho^{-}\biggl(\frac{{T}_{k,k}-\mathrm{E}{T}_{k,k}}{\beta\delta_{k}\sqrt {k}} ,\frac{{T}_{l,l}-\mathrm{E}{T}_{l,l}-({T}_{2k,l}-\mathrm{E}{T}_{2k,l})}{\beta \delta_{l}\sqrt{l}}\biggr) \\ &{}\cdot \biggl\Vert \frac{{T}_{k,k}-\mathrm{E}{T}_{k,k}}{\beta\delta_{k}\sqrt{k}} \biggr\Vert _{2,1}\cdot \biggl\Vert \frac {{T}_{l,l}-\mathrm{E}{T}_{l,l}-({T}_{2k,l}-\mathrm{E}{T}_{2k,l})}{\beta \delta_{l}\sqrt{l}} \biggr\Vert _{2,1}\biggr) \\ \leq& C\rho^{-}(k) \biggl(\operatorname{Var}\biggl(\frac{{T}_{k,k}-\mathrm{E}{T}_{k,k}}{\beta \delta_{k}\sqrt{k}} \biggr)\biggr)^{\frac{1}{2}}\cdot\biggl(\operatorname{Var} \biggl( \frac {{T}_{l,l}-\mathrm{E}{T}_{l,l}-({T}_{2k,l}-\mathrm{E}{T}_{2k,l})}{\beta \delta_{l}\sqrt{l}}\biggr)\biggr)^{\frac{1}{2}} \\ &{}+8\rho^{-}(k)\cdot \biggl\Vert \frac{{T}_{k,k}-\mathrm{E}{T}_{k,k}}{\beta \delta_{k}\sqrt{k}} \biggr\Vert _{2,1}\cdot \biggl\Vert \frac{{T}_{l,l}-\mathrm{E}{T}_{l,l}-({T}_{2k,l}-\mathrm{E}{T}_{2k,l})}{\beta \delta_{l}\sqrt{l}} \biggr\Vert _{2,1}. \end{aligned}$$

By $\|X\|_{2,1}\leq r/(r-2)\|X\|_{r}$, $r>2$ (see p. 254 of [10] or p. 251 of [13]), Minkowski inequality, Lemma 2.2, and the Hölder inequality, we get

$$\begin{aligned} \biggl\Vert \frac{{T}_{k,k}-\mathrm{E}{T}_{k,k}}{\beta \delta_{k}\sqrt{k}} \biggr\Vert _{2,1} \leq& \frac{r}{r-2} \biggl\Vert \frac {{T}_{k,k}-\mathrm{E}{T}_{k,k}}{\beta\delta_{k}\sqrt{k}} \biggr\Vert _{r} \\ =& \frac{r}{r-2}\frac{1}{\beta\delta_{k}\sqrt{k}}\bigl(\mathrm{E} \vert {T}_{k,k}-\mathrm{E} {T}_{k,k} \vert ^{r} \bigr)^{\frac{1}{r}} \\ \leq&\frac{C}{\sqrt{k}}\Biggl(\sum_{i=1}^{k} \mathrm{E}|\bar {Y}_{ki}|^{r}+\Biggl(\sum _{i=1}^{k} \mathrm{E}\bar{Y}_{ki}^{2} \Biggr)^{r/2}\Biggr)^{1/r} \\ \leq&\frac{C}{\sqrt {k}} \bigl(k+k^{r/2}\bigr)^{1/r}\leq C, \end{aligned}$$

similarly

$$\biggl\Vert \frac{{T}_{l,l}-\mathrm{E}{T}_{l,l}-({T}_{2k,l}-\mathrm{E}{T}_{2k,l})}{\beta \delta_{l}\sqrt{l}} \biggr\Vert _{2,1}\leq C. $$

Hence

9 $$ I_{2}\leq C\rho^{-}(k). $$

Combining with (7)–(9), (3) holds, and by (a_4_), Lemma 2.4, (6) holds, then (4) is true.

Secondly, we prove (5); for $\forall k \geq1$, $\eta_{k}=f({\bar {V}_{k,1}^{2}}/({k\delta_{k,1}^{2}}))-\mathrm{E}(f({\bar {V}_{k,1}^{2}}/({k\delta_{k,1}^{2}})))$, we have

10 $$\begin{aligned} \vert \mathrm{E}\eta_{k}\eta_{l} \vert =& \biggl\vert \operatorname{Cov}\biggl(f\biggl(\frac{\bar {V}_{k,1}^{2}}{k\delta_{k,1}^{2}}\biggr),f\biggl( \frac{\bar{V}_{l,1}^{2}}{l\delta _{l,1}^{2}}\biggr) \biggr) \biggr\vert \\ \leq& \biggl\vert \operatorname{Cov}\biggl(f\biggl(\frac{\bar{V}_{k,1}^{2}}{k\delta _{k,1}^{2}} \biggr),f\biggl(\frac{\bar{V}_{l,1}^{2}}{l\delta_{l,1}^{2}}\biggr) - f \biggl(\frac{\sum_{i=2k+1}^{l}\bar{Y}_{l,i}^{2}I({Y}_{i}\geq 0)}{l\delta_{l,1}^{2}}\biggr) \biggr) \biggr\vert \\ &{}+ \biggl\vert \operatorname{Cov}\biggl(f\biggl( \frac{\bar{V}_{k,1}^{2}}{k\delta_{k,1}^{2}}\biggr),f \biggl(\frac{\sum_{i=2k+1}^{l}\bar{Y}_{l,i}^{2}I({Y}_{i}\geq0)}{l\delta _{l,1}^{2}}\biggr)\biggr) \biggr\vert \\ =&J_{1}+J_{2}, \end{aligned}$$

by the property of *f*, we know

11 $$ J_{1}\leq C\Biggl(\mathrm{E}\Biggl(\sum_{i=1}^{2k} \bar{Y}_{ki}^{2}\mathrm{I}({Y}_{i}\geq 0)\Biggr)\Big/l \Biggr)\leq C\biggl(\frac{k}{l}\biggr). $$

Now we estimate $J_{2}$,

$$\begin{aligned} \operatorname{Var} \biggl(\frac{\bar{V}_{k,1}^{2}}{k\delta _{k,1}^{2}} \biggr) =& \operatorname{Var} \biggl(\frac{\sum_{i=1}^{k}\bar {Y}_{ki}^{2}\mathrm{I}({Y}_{i}\geq0)}{k\delta_{k,1}^{2}} \biggr) \\ \leq& \frac{C}{k^{2}} \mathrm{E}\Biggl(\sum_{i=1}^{k} \bar{Y}_{ki}^{2}\mathrm{I}({Y}_{i}\geq0) \Biggr)^{2} \\ =&\frac{C}{k^{2}} \mathrm{E} \Biggl(\sum_{i=1}^{k} \bar{Y}_{ki}^{2}\mathrm{I}({Y}_{i}\geq0)-\mathrm{E} \Biggl(\sum_{i=1}^{k}\bar{Y}_{ki}^{2} \mathrm{I}({Y}_{i}\geq0)\Biggr) +\mathrm{E}\Biggl(\sum _{i=1}^{k}\bar{Y}_{ki}^{2} \mathrm{I}({Y}_{i}\geq0)\Biggr) \Biggr)^{2} \\ \leq& \frac{C}{k^{2}} \mathrm{E} \Biggl(\sum_{i=1}^{k} \bigl(\bar {Y}_{ki}^{2}\mathrm{I}({Y}_{i}\geq0)- \mathrm{E}\bigl(\bar{Y}_{ki}^{2}\mathrm{I}({Y}_{i} \geq0)\bigr)\bigr) \Biggr)^{2} \\ &{}+\frac{C}{k^{2}} \Biggl(\sum _{i=1}^{k}\mathrm{E}\bigl(\bar{Y}_{ki}^{2} \mathrm{I}({Y}_{i}\geq0)\bigr)\Biggr)^{2} \\ \leq&\frac{C}{k^{2}}\sum_{i=1}^{k} \mathrm{E}\bar{Y}_{ki}^{4}\mathrm{I}({Y}_{i} \geq0)+\frac{C}{k^{2}}\bigl(k\mathrm{E}\bigl(\bar{Y}_{k1}^{2} \mathrm{I}({Y}_{1}\geq0)\bigr)\bigr)^{2} \\ \leq&\frac{C}{k^{2}}\sum_{i=1}^{k} \mathrm{E}k(Y_{i} )^{2}\leq C, \end{aligned}$$

and similarly $\operatorname{Var}(\sum_{i=2k+1}^{l}\bar{Y}_{li}^{2}\mathrm{I}({Y}_{i}\geq0)/ (l\delta_{l,1}^{2}))\leq C$. On the other hand, we have

$$\begin{aligned} \biggl\Vert \frac{\bar{V}_{k,1}^{2}}{k\delta _{k,1}^{2}} \biggr\Vert _{2,1} \leq& \frac{r}{r-2}\cdot\frac{C}{k}\bigl(\mathrm{E} \bigl\vert \bar {V}_{k,1}^{2} \bigr\vert ^{r} \bigr)^{1/r} \\ \leq& \frac{C}{k}\Biggl(\mathrm{E} \Biggl\vert \sum _{i=1}^{k}\bigl(\bar {Y}_{ki}^{2} \mathrm{I}({Y}_{i}\geq0)- \mathrm{E}\bigl(\bar{Y}_{ki}^{2} \mathrm{I}({Y}_{i}\geq0)\bigr)\bigr) \Biggr\vert ^{r} + \Biggl\vert \sum_{i=1}^{k} \mathrm{E}\bigl( \bar{Y}_{ki}^{2}\mathrm{I}({Y}_{i}\geq 0)\bigr) \Biggr\vert ^{r}\Biggr)^{1/r} \\ \leq&\frac{C}{k}\Biggl(\sum_{i=1}^{k} \mathrm{E} \bigl\vert \bigl(\bar {Y}_{ki}^{2} \mathrm{I}({Y}_{i}\geq0)- \mathrm{E}\bigl(\bar{Y}_{ki}^{2} \mathrm{I}({Y}_{i}\geq0)\bigr)\bigr) \bigr\vert ^{r} \\ &{}+ \Biggl( \sum_{i=1}^{k}\mathrm{E} \bigl( \bar{Y}_{ki}^{2}\mathrm{I}({Y}_{i}\geq0)- \mathrm{E}\bigl(\bar{Y}_{ki}^{2}\mathrm{I}({Y}_{i} \geq0)\bigr)\bigr)^{2}\Biggr)^{r/2}\Biggr)^{1/r} \\ &{}+\frac{C}{k} \Biggl\vert \sum_{i=1}^{k} \mathrm{E}\bigl(\bar{Y}_{ki}^{2}\mathrm{I}({Y}_{i} \geq0)\bigr) \Biggr\vert \\ \leq& \frac{C}{k}\Biggl(\sum_{i=1}^{k} \mathrm{E} \bigl\vert \bar {Y}_{ki}^{2} \mathrm{I}({Y}_{i}\geq0) \bigr\vert ^{r} + \Biggl(\sum _{i=1}^{k} \mathrm{E} \bigl\vert \bar{Y}_{ki}^{2}\mathrm{I}({Y}_{i}\geq0) \bigr\vert ^{2}\Biggr)^{r/2}\Biggr)^{1/r} \\ &{}+ \frac{C}{k} \bigl\vert k \mathrm{E}\bigl(\bar{Y}_{k1}^{2} \mathrm{I}({Y}_{1}\geq0)\bigr) \bigr\vert \\ \leq&\frac{C}{k}\Biggl(\sum_{i=1}^{k} \mathrm{E} \vert \sqrt {k} {Y}_{i} \vert ^{r}+\Biggl( \sum_{i=1}^{k} \mathrm{E} \vert \sqrt {k} {Y}_{i} \vert ^{2}\Biggr)^{r/2} \Biggr)^{1/r} +C_{1} \\ \leq& \frac{C}{k}\bigl(k^{1+{r/2}}+k^{r} \bigr)^{1/r}+C_{1}\leq C, \end{aligned}$$

similarly

$$\Biggl\Vert \sum_{i=2k+1}^{l} \bar{Y}_{li}^{2}\mathrm{I}({Y}_{i}\geq0)/ \bigl(l \delta_{l,1}^{2}\bigr) \Biggr\Vert _{2,1}\leq C. $$

Thus, by Lemma 2.3, we have

12 $$\begin{aligned} J_{2} \leq& C\biggl\{ -\operatorname{Cov}\biggl(\frac{\bar{V}_{k,1}^{2}}{k\delta _{k,1}^{2}}, \frac{\sum_{i=2k+1}^{l}\bar{Y}_{li}^{2}\mathrm{I}({Y}_{i}\geq 0)}{l\delta_{l,1}^{2}}\biggr) \\ &{}+8\rho^{-}\biggl( \frac{\bar {V}_{k,1}^{2}}{k\delta_{k,1}^{2}},\frac{\sum_{i=2k+1}^{l}\bar {Y}_{li}^{2}\mathrm{I}({Y}_{i}\geq0)}{l\delta_{l,1}^{2}} \biggr) \cdot \biggl\Vert \frac{\bar{V}_{k,1}^{2}}{k\delta_{k,1}^{2}} \biggr\Vert _{2,1}\cdot \biggl\Vert \frac{\sum_{i=2k+1}^{l}\bar{Y}_{li}^{2}\mathrm{I}({Y}_{i}\geq0)}{l\delta _{l,1}^{2}} \biggr\Vert _{2,1}\biggr\} \\ \leq& C\biggl\{ \rho^{-}(k) \biggl(\operatorname{Var}\biggl( \frac{\bar {V}_{k,1}^{2}}{k\delta_{k,1}^{2}}\biggr)\biggr)^{1/2}\cdot \operatorname{Var}\biggl( \frac {\sum_{i=2k+1}^{l}\bar{Y}_{li}^{2}\mathrm{I}({Y}_{i}\geq0)}{l\delta _{l,1}^{2}}\biggr)^{1/2} \\ &{}+\rho^{-}(k)\cdot \biggl\Vert \frac{\bar{V}_{k,1}^{2}}{k\delta _{k,1}^{2}} \biggr\Vert _{2,1}\cdot \biggl\Vert \frac{\sum_{i=2k+1}^{l}\bar {Y}_{li}^{2}\mathrm{I}({Y}_{i}\geq0)}{l\delta_{l,1}^{2}} \biggr\Vert _{2,1} \biggr\} \\ \leq&C\rho^{-}(k), \end{aligned}$$

hence, combining with (11) and (12), (3) holds, and by Lemma 2.4, (5) holds. □

## Proof of Theorem 1

Let $C_{k,i}= \frac{S_{k,i}}{(k-1)\mu}$, hence, (2) is equivalent to

13 $$ \lim_{n\rightarrow\infty}\frac{1}{D_{n}}\sum_{k=1}^{n} d_{k}\mathrm{I}\Biggl(\frac{\mu}{\beta V_{k}}\sum _{i=1}^{k}\log C_{k,i}\leq{x}\Biggr)= \Phi(x) \quad \mbox{a.s.} $$

So we only need to prove (13), for a fixed *k*, $1\leq k\leq n$ and $\forall\varepsilon>0$; we have

$$\lim_{k\rightarrow\infty}P\Biggl\{ \bigcup_{m=k}^{\infty} \biggl( \biggl\vert \frac {X_{i}}{m} \biggr\vert \geq\varepsilon\biggr) \Biggr\} =\lim_{k\rightarrow\infty}P\biggl\{ \biggl\vert \frac{X_{i}}{k} \biggr\vert \geq\varepsilon \biggr\} =\lim_{k\rightarrow\infty}P\bigl\{ | X_{1}|\geq \varepsilon k\bigr\} =0, $$

therefore, by Theorem 1.5.2 in [14], we have

$$\frac{X_{i}}{k}\rightarrow0\quad \mbox{a.s. }k\rightarrow\infty, $$

on the unanimous establishment of *i*.

By Lemma 2.1, for some $\frac{4}{3}< p<2 $, and enough large *k*, we have

$$\begin{aligned} \sup_{1\leq i\leq k} \vert C_{k,i}-1 \vert \leq& \biggl\vert \frac{\sum_{j=1}^{k}(X_{j}-\mu)}{(k-1)\mu} \biggr\vert +\sup_{1\leq i\leq k} \biggl\vert \frac{X_{i}}{(k-1)\mu} \biggr\vert +\frac {1}{k-1} \\ \leq& \biggl\vert \frac{S_{k}-k\mu}{k^{\frac{1}{p}}}\cdot\frac{k^{\frac {1}{p}}}{(k-1)\mu} \biggr\vert \leq Ck^{\frac{1}{p}-1}, \end{aligned}$$

by $\log(1+x)=x+O(x^{2})$, $x\rightarrow0$, we get

$$\begin{aligned} &\Biggl\vert \frac{\mu}{\beta\delta_{k}\sqrt{(1\pm\varepsilon)k}}\sum_{i=1}^{k} \ln{C}_{k,i}-\frac{\mu}{\beta\delta_{k}\sqrt{(1\pm\varepsilon )k}}\sum_{i=1}^{k}(C_{k,i}-1) \Biggr\vert \\ &\quad \leq\frac{{C}\mu}{\beta\delta_{k}\sqrt{(1\pm\varepsilon)k}}\sum_{i=1}^{k}(C_{k,i}-1)^{2} \\ &\quad \leq\frac{C}{\sqrt{k}}k^{\frac{2}{p}-1}\rightarrow0\quad \mbox{a.s.}, k \rightarrow \infty, \end{aligned}$$

and then, for $\delta>0$ and every *ω*, there exists $k_{0}=k_{0}(\omega,\delta,x)$; when $k>k_{0}$, we have

14 $$\begin{aligned} &\mathrm{I}\Biggl\{ \frac{\mu}{\beta\delta_{k}\sqrt{(1\pm\varepsilon)k}}\sum_{i=1}^{k}(C_{k,i}-1) \leq{x}-\delta\Biggr\} \\ &\quad \leq\mathrm{I}\Biggl\{ \frac{\mu}{\beta\delta_{k}\sqrt{(1\pm\varepsilon )k}}\sum _{i=1}^{k}\log{C}_{k,i}\leq{x}\Biggr\} \\ &\quad \leq\mathrm{I}\Biggl\{ \frac{\mu}{\beta\delta_{k}\sqrt{(1\pm\varepsilon )k}}\sum_{i=1}^{k}(C_{k,i}-1) \leq{x}+\delta\Biggr\} , \end{aligned}$$

under the condition $|X_{i}-\mu|\leq\sqrt{k}$, $1\leq i\leq k$, we have

15 $$ \mu\sum_{i=1}^{k}(C_{k,i}-1)=\sum _{i=1}^{k}\frac{S_{k,i}-(k-1)\mu }{k-1}=\sum _{i=1}^{k}Y_{i}=\sum _{i=1}^{k}\bar{Y}_{ki}={T}_{k,i}, $$

furthermore, by (14) and (15), for any given $0<\varepsilon<1$, $\delta >0$, when $k>k_{0}$, we obtain

$$\begin{aligned}& \mathrm{I}\Biggl(\frac{\mu}{\beta V_{k}}\sum_{i=1}^{k} \log C_{k,i}\leq x\Biggr) \\& \quad \leq\mathrm{I}\biggl(\frac{{T}_{k,i}}{\delta_{k}\beta\sqrt{k(1+\varepsilon )}}\leq x+ \delta\biggr) +\mathrm{I}\bigl(\bar{V}_{k}^{2}>(1+ \varepsilon)k\delta_{k}^{2}\bigr) \\& \qquad {}+\mathrm{I}\Biggl(\bigcup _{i=1}^{k}\bigl( \vert X_{i}-\mu \vert >\sqrt{k}\bigr)\Biggr),\quad x\geq0, \\& \mathrm{I}\Biggl(\frac{\mu}{\beta V_{k}}\sum_{i=1}^{k} \log C_{k,i}\leq x\Biggr) \\& \quad \leq\mathrm{I}\biggl(\frac{{T}_{k,i}}{\delta_{k}\beta\sqrt{k(1-\varepsilon )}} \leq x+ \delta\biggr) +\mathrm{I}\bigl(\bar{V}_{k}^{2}< (1- \varepsilon)k\delta_{k}^{2}\bigr) \\& \qquad {}+\mathrm{I}\Biggl(\bigcup _{i=1}^{k}\bigl( \vert X_{i}-\mu \vert >\sqrt{k}\bigr)\Biggr),\quad x< 0, \\& \mathrm{I}\Biggl(\frac{\mu}{\beta V_{k}}\sum_{i=1}^{k} \log C_{k,i}\leq x\Biggr) \\& \quad \geq\mathrm{I}\biggl(\frac{{T}_{k,i}}{\delta_{k}\beta\sqrt{k(1-\varepsilon )}}\leq x- \delta\biggr) -\mathrm{I}\bigl(\bar{V}_{k}^{2}< (1- \varepsilon)k\delta_{k}^{2}\bigr) \\& \qquad {}-\mathrm{I}\Biggl(\bigcup _{i=1}^{k}\bigl( \vert X_{i}-\mu \vert >\sqrt{k}\bigr)\Biggr),\quad x\geq0, \\& \mathrm{I}\Biggl(\frac{\mu}{\beta V_{k}}\sum_{i=1}^{k} \log C_{k,i}\leq x\Biggr) \\& \quad \geq\mathrm{I}\biggl(\frac{{T}_{k,i}}{\delta_{k}\beta\sqrt{k(1+\varepsilon )}}\leq x- \delta\biggr) -\mathrm{I}\bigl(\bar{V}_{k}^{2}>(1+ \varepsilon)k\delta_{k}^{2}\bigr) \\& \qquad {}-\mathrm{I}\Biggl(\bigcup _{i=1}^{k}\bigl( \vert X_{i}-\mu \vert >\sqrt{k}\bigr)\Biggr),\quad x< 0. \end{aligned}$$

Therefore, to prove (13), for any $0<\varepsilon<1$, $\delta_{1}>0$, it suffices to prove

16 $$\begin{aligned}& \lim_{n\rightarrow\infty}\frac{1}{D_{n}}\sum_{k=1}^{n}d_{k} \mathrm{I} \biggl( \frac{{T}_{k,i}}{\beta\delta_{k}\sqrt{k}}\leq\sqrt{1\pm\varepsilon }x\pm \delta_{1} \biggr)=\Phi(\sqrt{1\pm\varepsilon}x\pm\delta_{1}) \quad \mbox{a.s.} , \end{aligned}$$

17 $$\begin{aligned}& \lim_{n\rightarrow\infty}\frac{1}{D_{n}}\sum_{k=1}^{n}d_{k} \mathrm{I}\Biggl(\bigcup_{i=1}^{k}\bigl( \vert X_{i}-\mu \vert >\sqrt{k}\bigr)\Biggr)=0\quad \mbox{a.s.} , \end{aligned}$$

18 $$\begin{aligned}& \lim_{n\rightarrow\infty}\frac{1}{D_{n}}\sum_{k=1}^{n}d_{k} \mathrm{I} \bigl(\bar{V}_{k}^{2}>(1+\varepsilon)k \delta_{k}^{2}\bigr)=0 \quad \mbox{a.s.} , \end{aligned}$$

19 $$\begin{aligned}& \lim_{n\rightarrow\infty}\frac{1}{D_{n}}\sum_{k=1}^{n}d_{k} \mathrm{I} \bigl(\bar{V}_{k}^{2}< (1-\varepsilon)k \delta_{k}^{2}\bigr)=0 \quad \mbox{a.s.} \end{aligned}$$

Firstly, we prove (16), by $\mathrm{E}(Y^{2})<\infty$, we know $\lim_{x\rightarrow\infty}x^{2}P(|Y|>x)=0$, and by $\mathrm{E}(Y)=0$, it follows that

$$\begin{aligned} \bigl\vert \mathrm{E}({T}_{k,i}) \bigr\vert =& \Biggl\vert \mathrm{E}\Biggl(\sum_{i=1}^{k}\bar {Y}_{ki}\Biggr) \Biggr\vert = \vert k\mathrm{E} \bar{Y}_{k1} \vert \\ \leq& k \bigl\vert \mathrm{E}\bigl(Y\mathrm{I}\bigl( \vert Y \vert > \sqrt{k}\bigr)\bigr) \bigr\vert +k^{\frac{3}{2}}\mathrm{E}\bigl(\mathrm{I} \bigl( \vert Y \vert >\sqrt{k}\bigr)\bigr) \\ \leq&\sqrt{k}\mathrm{E}\bigl(Y^{2}\mathrm{I}\bigl( \vert Y \vert >\sqrt{k}\bigr)\bigr)+k^{\frac {3}{2}}P\bigl( \vert Y \vert >\sqrt{k} \bigr)=o(\sqrt{k}), \end{aligned}$$

so, combining with $\delta_{k}^{2}\rightarrow\mathrm{E}(Y^{2})<\infty$, for any $\alpha>0$, when $k\rightarrow\infty$, we have

$$\begin{aligned} &\mathrm{I} \biggl(\frac{{T}_{k,i}-E{T}_{k,i}}{\beta\delta _{k}\sqrt{k}}\leq\sqrt{1\pm\varepsilon} x\pm \delta_{1}-\alpha \biggr) \\ &\quad \leq \mathrm{I} \biggl(\frac{{T}_{k,i}}{\beta\delta_{k}\sqrt{k}} \leq\sqrt {1\pm\varepsilon}x\pm\delta_{1} \biggr) \\ &\quad \leq \mathrm{I} \biggl(\frac{{T}_{k,i}-E{T}_{k,i}}{\beta\delta_{k}\sqrt{k}}\leq \sqrt{1\pm\varepsilon}x\pm \delta_{1}+\alpha \biggr), \end{aligned}$$

thus, by (4), we get

20 $$\begin{aligned}& \begin{aligned}[b] &\lim_{n\rightarrow\infty}\frac{1}{D_{n}}\sum _{k=1}^{n}d_{k} \mathrm{I} \biggl( \frac{{T}_{k,i}}{\beta\delta_{k}\sqrt {k}}\leq\sqrt{1\pm\varepsilon}x\pm\delta_{1} \biggr) \\ &\quad \geq \lim_{n\rightarrow\infty}\frac{1}{D_{n}}\sum _{k=1}^{n}d_{k} \mathrm{I} \biggl( \frac{{T}_{k,i}-E{T}_{k,i}}{\beta\delta_{k}\sqrt{k}}\leq \sqrt{1\pm\varepsilon}x\pm\delta_{1}-\alpha \biggr) \\ &\quad \rightarrow\Phi(\sqrt{1\pm\varepsilon}x\pm\delta_{1}-\alpha), \end{aligned} \end{aligned}$$

21 $$\begin{aligned}& \begin{aligned}[b] &\lim_{n\rightarrow\infty}\frac{1}{D_{n}}\sum _{k=1}^{n}d_{k} \mathrm{I} \biggl( \frac{{T}_{k,i}}{\beta\delta_{k}\sqrt {k}}\leq\sqrt{1\pm\varepsilon}x\pm\delta_{1} \biggr) \\ &\quad \leq\lim_{n\rightarrow\infty}\frac{1}{D_{n}}\sum _{k=1}^{n}d_{k}\mathrm{I} \biggl( \frac{{T}_{k,i}-E{T}_{k,i}}{\beta\delta_{k}\sqrt{k}}\leq\sqrt {1\pm\varepsilon} x\pm\delta_{1}+\alpha \biggr) \\ &\quad \rightarrow \Phi(\sqrt{1\pm\varepsilon}x\pm\delta_{1}+\alpha)\quad \mbox{a.s.}, \end{aligned} \end{aligned}$$

letting $\alpha\rightarrow0$ in (20) and (21), (16) holds.

Now, we prove (17); by $\mathrm{E}(Y^{2})<\infty$, we know $\lim_{x\rightarrow\infty}x^{2}P(|Y|>x)=0$, such that

$$\mathrm{E}\mathrm{I}\Biggl(\bigcup_{i=1}^{k} \bigl( \vert Y_{i} \vert >\sqrt{k}\bigr)\Biggr)\leq\sum _{i=1}^{k}P\bigl( \vert Y_{i} \vert > \sqrt{k}\bigr)\leq kP\bigl( \vert Y \vert >\sqrt{k}\bigr)\rightarrow 0,\quad k \rightarrow\infty, $$

by the Toeplitz lemma, we get

22 $$ \lim_{n\rightarrow\infty}\frac{1}{D_{n}}\sum_{k=1}^{n}d_{k} \mathrm{E}\mathrm{I}\Biggl(\bigcup_{i=1}^{k} \bigl( \vert Y_{i} \vert >\sqrt{k}\bigr)\Biggr)\rightarrow0\quad \mbox{a.s.}, $$

hence, to prove (17), it suffices to prove

23 $$ \lim_{n\rightarrow\infty}\frac{1}{D_{n}}\sum_{k=1}^{n}d_{k} \Biggl(\mathrm{I}\Biggl(\bigcup_{i=1}^{k} \bigl( \vert Y_{i} \vert >\sqrt{k}\bigr)\Biggr)-\mathrm{E} \Biggl[ \mathrm{I}\Biggl(\bigcup_{i=1}^{k}\bigl( \vert Y_{i} \vert >\sqrt{k}\bigr)\Biggr) \Biggr] \Biggr) \rightarrow0\quad \mbox{a.s.}, $$

writing

$$\mathscr {Z}_{k}= \mathrm{I}\Biggl(\bigcup _{i=1}^{k}\bigl( \vert Y_{i} \vert > \sqrt{k}\bigr)\Biggr)-\mathrm{E} \Biggl[\mathrm{I}\Biggl(\bigcup _{i=1}^{k}\bigl( \vert Y_{i} \vert > \sqrt{k}\bigr)\Biggr) \Biggr], $$

for every $0\leq2k< l$, so by the definition of $\rho^{-}$-mixing sequence, we have

$$\begin{aligned} \mathrm{E} \vert \mathscr {Z}_{k}\mathscr {Z}_{l} \vert =& \Biggl\vert \operatorname{Cov}\Biggl(\mathrm{I}\Biggl(\bigcup _{i=1}^{k}\bigl( \vert Y_{i} \vert > \sqrt{k}\bigr)\Biggr),\mathrm{I}\Biggl(\bigcup_{i=1}^{l} \bigl( \vert Y_{i} \vert >\sqrt{l}\bigr)\Biggr) \Biggr) \Biggr\vert \\ \leq& \Biggl\vert \operatorname{Cov}\Biggl(\mathrm{I}\Biggl(\bigcup _{i=1}^{k}\bigl( \vert Y_{i} \vert > \sqrt {k}\bigr)\Biggr),\mathrm{I}\Biggl(\bigcup_{i=1}^{l} \bigl( \vert Y_{i} \vert >\sqrt{l}\bigr)\Biggr)-\mathrm{I}\Biggl( \bigcup_{i=2k+1}^{l}\bigl( \vert Y_{i} \vert >\sqrt{l}\bigr)\Biggr) \Biggr) \Biggr\vert \\ &{}+ \Biggl\vert \operatorname{Cov}\Biggl(\mathrm{I}\Biggl(\bigcup _{i=1}^{k}\bigl( \vert Y_{i} \vert > \sqrt {k}\bigr)\Biggr),\mathrm{I}\Biggl(\bigcup_{i=2k+1}^{l} \bigl( \vert Y_{i} \vert >\sqrt{l}\bigr)\Biggr) \Biggr) \Biggr\vert \\ \leq&\mathrm{E} \Biggl\vert \mathrm{I}\Biggl(\bigcup _{i=1}^{l}\bigl( \vert Y_{i} \vert > \sqrt{l}\bigr)\Biggr)-\mathrm{I}\Biggl(\bigcup_{i=2k+1}^{l} \bigl( \vert Y_{i} \vert >\sqrt{l}\bigr)\Biggr) \Biggr\vert \\ &{}+ \rho^{-}(k)\sqrt{\operatorname{Var}\Biggl(\mathrm{I} \Biggl(\bigcup_{i=1}^{k}\bigl( \vert Y_{i} \vert >\sqrt {k}\bigr)\Biggr)\Biggr)\operatorname{Var}\Biggl( \mathrm{I}\Biggl(\bigcup_{i=2k+1}^{l}\bigl( \vert Y_{i} \vert >\sqrt{l}\bigr)\Biggr)\Biggr)} \\ \leq&\mathrm{E}\Biggl[\mathrm{I}\Biggl(\bigcup_{i=1}^{2k} \bigl( \vert Y_{i} \vert >\sqrt{l}\bigr)\Biggr) \Biggr]+C \rho^{-}(k) \\ \leq&\sum_{i=1}^{k}P\bigl( \vert Y_{i} \vert >\sqrt{l}\bigr)+C\rho^{-}(k) \\ \leq& kP\bigl( \vert Y \vert >\sqrt{l}\bigr)+C\rho^{-}(k) \\ \leq& C\biggl(\frac{k}{l}+\rho^{-}(k)\biggr), \end{aligned}$$

so by Lemma 2.4, (23) holds. And combining with (22), we know that (17) holds.

Next, we prove (18); by $\mathrm{E}(\bar{V}_{k}^{2})=k\delta_{k}^{2}$, $\bar{V}_{k}^{2}=\bar{V}_{k,1}^{2}+\bar{V}_{k,2}^{2}$, $\mathrm{E}(\bar {V}_{k,l}^{2})=k\delta_{k,l}^{2}$, and $\delta_{k,1}^{2}\leq\delta_{k}^{2}$, $l=1,2$, we have

$$\begin{aligned} \mathrm{I}\bigl(\bar{V}_{k}^{2}>(1+\varepsilon)k \delta_{k}^{2}\bigr) =&\mathrm{I}\bigl(\bar {V}_{k}^{2}-\mathrm{E}\bigl(\bar{V}_{k}^{2} \bigr)>\varepsilon k\delta_{k}^{2}\bigr) \\ \leq& \mathrm{I}\bigl(\bar{V}_{k,1}^{2}-\mathrm{E}\bigl(\bar {V}_{k,1}^{2}\bigr)>\varepsilon k\delta_{k}^{2}/2 \bigr)+\mathrm{I}\bigl(\bar {V}_{k,2}^{2}-\mathrm{E}\bigl( \bar{V}_{k,2}^{2}\bigr)>\varepsilon k\delta _{k}^{2}/2\bigr) \\ \leq& \mathrm{I}\biggl(\bar{V}_{k,1}^{2}> \biggl(1+ \frac{\varepsilon}{2}\biggr)k\delta _{k,1}^{2} \biggr)+ \mathrm{I} \biggl(\bar{V}_{k,2}^{2}> \biggl(1+\frac{\varepsilon}{2}\biggr)k \delta_{k,2}^{2}\biggr), \end{aligned}$$

therefore, by the arbitrariness of $\varepsilon>0$, to prove (18), it suffices to prove

24 $$ \lim_{n\rightarrow\infty}\frac{1}{D_{n}}\sum_{k=1}^{n}d_{k} \mathrm{I} \biggl(\bar{V}_{k,l}^{2}>\biggl(1+\frac{\varepsilon}{2} \biggr)k\delta_{k,l}^{2}\biggr)=0\quad \mbox{a.s. } l=1,2, $$

when $l=1$, for given $\varepsilon>0$, let *f* be a bounded function with bounded continuous derivative such that

25 $$ \mathrm{I}(x>1+\varepsilon)\leq f(x)\leq\mathrm{I}\biggl(x>1+\frac{\varepsilon }{2} \biggr), $$

under the condition

$$\mathrm{E}\bigl(\bar{V}_{k,1}^{2}\bigr)=k \delta_{k,1}^{2},\qquad \mathrm{E}\bigl(Y^{2}\bigr)< \infty,\qquad \mathrm{E}\bigl(Y^{2}\mathrm{I}(Y\geq0)\bigr)>0, $$

by the Markov inequality, and Lemma 2.2, we get

26 $$\begin{aligned} &P\biggl(\bar{V}_{k,1}^{2}>\biggl(1+\frac{\varepsilon}{2}\biggr)k \delta _{k,1}^{2}\biggr) \\ &\quad =P\biggl(\bar{V}_{k,1}^{2}- \mathrm{E}\bigl(\bar{V}_{k,1}^{2}\bigr)>\frac {\varepsilon}{2} k \delta_{k,1}^{2}\biggr) \\ &\quad \leq C\frac{\mathrm{E}(\bar{V}_{k,1}^{2}-\mathrm{E}(\bar {V}_{k,1}^{2}))^{2}}{k^{2}} \leq C\frac{\sum_{i=1}^{k}\mathrm{E}(\bar{Y}_{ki}^{2}\mathrm{I}(\bar {Y}_{ki}\geq0))^{2}}{k^{2}} \\ &\quad \leq C\frac{\mathrm{E}\bar{Y}_{k1}^{4}\mathrm{I}(\bar{Y}_{k1}\geq0)}{k} \leq C\frac{\mathrm{E}Y^{4}\mathrm{I}(0\leq Y\leq\sqrt{k})+k^{2}P(Y>\sqrt{k})}{k}, \end{aligned}$$

because $\mathrm{E}(Y^{2})<\infty$ implies $\lim_{x\rightarrow\infty }x^{2}P(|Y|>x)=0$, we have

$$\begin{aligned} \mathrm{E}Y^{4}\mathrm{I}(0\leq Y\leq\sqrt{k}) =& \int _{0}^{\infty}P\bigl( \vert Y \vert \mathrm{I}(0 \leq Y\leq\sqrt{k})\geq t \bigr)4t^{3}\,dt \\ \leq&C \int_{0}^{\sqrt{k}}P\bigl( \vert Y \vert \geq t \bigr)t^{3}\,dt \\ =& \int_{0}^{\sqrt{k}}o(1)t\, dt =o(1)k, \end{aligned}$$

thus, combining with (26),

$$P\biggl(\bar{V}_{k,1}^{2}>\biggl(1+\frac{\varepsilon}{2}\biggr)k \delta _{k,1}^{2}\biggr)\rightarrow0 ,\quad k\rightarrow\infty. $$

Therefore, from (5), (25) and the Toeplitz lemma

$$\begin{aligned} 0 \leq& \frac{1}{D_{n}}\sum_{k=1}^{n}d_{k} \mathrm{I} \biggl(\bar{V}_{k,1}^{2}>\biggl(1+\frac{\varepsilon}{2} \biggr)k\delta_{k,1}^{2}\biggr) \\ \leq&\frac{1}{D_{n}}\sum_{k=1}^{n}d_{k}f \biggl(\frac{\bar {V}_{k,1}^{2}}{k\delta_{k,1}^{2}}\biggr) \\ =&\frac{1}{D_{n}}\sum_{k=1}^{n}d_{k} \mathrm{E} \biggl(f\biggl(\frac{\bar {V}_{k,1}^{2}}{k\delta_{k,1}^{2}}\biggr) \biggr) +\frac{1}{D_{n}} \sum_{k=1}^{n}d_{k} \biggl(f \biggl(\frac{\bar {V}_{k,1}^{2}}{k\delta_{k,1}^{2}}\biggr)-\mathrm{E}\biggl(f\biggl(\frac{\bar {V}_{k,1}^{2}}{k\delta_{k,1}^{2}} \biggr)\biggr) \biggr) \\ \leq&\frac{1}{D_{n}}\sum_{k=1}^{n}d_{k} \mathrm{E}\biggl(\mathrm{I} \biggl(\bar {V}_{k,1}^{2}>\biggl(1+ \frac{\varepsilon}{2}\biggr)k\delta_{k,1}^{2}\biggr)\biggr) + \frac{1}{D_{n}}\sum_{k=1}^{n}d_{k} \biggl(f\biggl(\frac{\bar {V}_{k,1}^{2}}{k\delta_{k,1}^{2}}\biggr)-\mathrm{E}\biggl(f\biggl( \frac{\bar {V}_{k,1}^{2}}{k\delta_{k,1}^{2}}\biggr)\biggr) \biggr) \\ =&\frac{1}{D_{n}}\sum_{k=1}^{n}d_{k}P \biggl(\bar{V}_{k,1}^{2}>\biggl(1+\frac {\varepsilon}{2}\biggr)k \delta_{k,1}^{2}\biggr) +\frac{1}{D_{n}}\sum _{k=1}^{n}d_{k} \biggl(f\biggl( \frac{\bar {V}_{k,1}^{2}}{k\delta_{k,1}^{2}}\biggr)-\mathrm{E}\biggl(f\biggl(\frac{\bar {V}_{k,1}^{2}}{k\delta_{k,1}^{2}}\biggr) \biggr) \biggr) \\ \rightarrow&0 \quad \mbox{a.s.}, k\rightarrow\infty, \end{aligned}$$

hence, (24) holds for $l=1$. Similarly, we can prove (24) for $l=2$, so (18) is true. By similar methods used to prove (18), we can prove (19), this completes the proof of Theorem 1.
